# A critical analysis of walking policy in Ireland and its contribution to both national and international development goals

**DOI:** 10.3389/fspor.2023.1125636

**Published:** 2023-03-01

**Authors:** Dylan Power, Barry Lambe, Niamh Murphy

**Affiliations:** ^1^ Centre for Health Behaviour Research, Department of Sport and Exercise Science, South East Technological University, Waterford, Ireland; ^2^ Get Ireland Walking, Mountaineering Ireland, Dublin, Ireland

**Keywords:** walking, content analysis, physical activity policy, pragmatic, sustainable development goals

## Abstract

**Introduction:**

Increasing population levels of walking holds benefits for public and planetary health. While individual level interventions to promote walking have been shown to be efficacious, upstream interventions such as policies harness the greatest potential for impact at the population level. However, little is known about the nature and presence of walking policy in Ireland and the extent to which it aligns to national and global goals. This paper aims to provide an overview of local and national walking policy in Ireland and to understand the potential of Irish walking policy to contribute to national and global targets.

**Methods:**

This study used multiple methods to provide a critical overview of walking policy. Firstly, a six-phase process was employed to conduct a content analysis of local and national walking policy in Ireland. Secondly, conceptual linkage exercises were conducted to assess the contribution of walking, and national walking policy in Ireland, to Ireland's National Strategic Outcomes and the United Nations Sustainable Development Goals.

**Results:**

Overall, half (*n* = 13) of the counties in the Republic of Ireland were found to have no local level walking policies. Results from the content analysis suggest that counties which had walking specific local level policies (*n* = 2) were outdated by almost two decades. Walking was identified to hold the potential to contribute to over half (*n* = 6) of Ireland's National Strategic Outcomes, and over half (*n* = 7) of the United Nations Sustainable Development Goals. Ireland's only national level walking specific policy, the Get Ireland Walking Strategy and Action Plan 2017–2020, was identified to potentially contribute to four of Ireland's National Strategic Outcomes and three United Nations Sustainable Development Goals.

**Discussion:**

Multidisciplinary action is required to update walking-related policy with embedded evaluation and governance mechanisms in all local walking systems. Furthermore, given sufficient collaboration across sectors, walking policy in Ireland has the potential to contribute to a wider breadth of national and global targets beyond the health, sport, tourism, and transport sectors.

## Introduction

The introduction of systems thinking in public health practice and research has provided stakeholders embedded within public health systems new perspectives on the interconnections between their own work, and the work of organisations from other sectors and disciplines in the system (1, 2). Many conceptual tools, such as applying a systems lens or “systems framing” have been adopted by researchers and practitioners to help stakeholders to develop a systems oriented view of the systems which they are embedded within (2, 3). Oftentimes, this involves asking stakeholders to “take a step back” and can offer stakeholders insight into the wider goals and systems that they influence/are influenced by. Physical activity (PA) policy researchers have begun to develop conceptual frameworks to assist with this notion of “zooming out” in order to understand the interconnections across policy sectors, disciplines and organisations involved in all stages of PA policy (4). At a global level, in 2015, the United Nations published the 2030 Agenda for Sustainable Development, which provides all member states of the United Nations a “shared blueprint for peace and prosperity for people and the planet” (5). At the core of the 2030 Agenda for Sustainable Development are 17 goals, termed the Sustainable Development Goals (SDGs). The SDGs require international, national, and local partnerships and policies to achieve high-level goals which aim to improve health, education, reduce inequalities, tackle climate change and end poverty across all United Nations member states (5).

The relationship between the SDGs and PA has garnered recent research attention. In a mixed methods paper, Salvo et al. (6) used agent-based modelling, a conceptual linkage exercise, and a scoping review of the literature to explore the synergy between at-scale PA promotion and the SDGs. Salvo and colleagues found at scale PA promotion may hold possible benefits for 15 SDGs (6). Moreover, Bauman (7) put forward an argument which outlines the potential for the sustainable development agenda to revitalise international PA promotion and research, by facilitating a broader, more systems-oriented view of the impacts of PA. Among many factors, the lack of upstream interventions, such as policies, promoting PA has led to stagnating physical inactivity levels globally and an evidence base congested with individual level, cross sectional studies (8, 9). The recent shift towards systems approaches to PA has begun to incorporate sectors beyond health, sport and transport in to conversations pertaining to PA (3, 10–12). However, ensuring PA policy and research decisions are transparent with global targets can supplement whole-of-systems approaches and can provide a new momentum to PA promotion (7).

Some organisations in the PA system have used the SDGs as a roadmap to determine the impact of their work and policies on the SDGs. For example, in a conceptual mapping exercise assessing the potential contribution of sports policies to the attainment of SDGs across countries in the Commonwealth, Sherry and colleagues (13) found direct links between some SDGs (SDG 3; SDG 4; SDG 10; and SDG 16) and actions within national sports policies across the Commonwealth. However, the findings also allowed Sherry and colleagues (13) to identify opportunities for sport policies to extend their scope and contribute to other SDG targets pertaining to much broader societal issues such as climate change (SDG 13). At a more granular level, Amosa and Lauff (14) determined the contribution of sport policies in Fiji and Samoa on the attainment of SDG goals through a similar conceptual mapping exercise. Amosa and Lauff found a list of context specific indicator datasets which can help monitor progress towards 132 of the 232 SDG indicators (14). Exercises such as those mentioned (6, 13, 14) can provide the opportunity to view the work of an organisation, or national level policies through an SDG lens. This, in turn, allows opportunities for data collection, intervention implementation, and policy development to be identified and informed. Although investment in promoting more walking and cycling has been suggested to be one of the “8 Investments that work for Physical Activity” (15), there is little known about how walking or cycling can specifically contribute to higher level goals.

Scotland's national walking promotion organisation, Paths for All, have made efforts to ensure alignment between the work conducted through the organisation at local and national level to global level objectives for PA outlined in the Global Action Plan for Physical Activity 2018–2030 (16, 17). In 2020, the Irish government have allocated €1 m per day to walking and cycling promotion and development (18), and there is an opportunity now to understand the potential contribution that increased walking levels may have on national and global targets. Get Ireland Walking, a national walking promotion organisation, was established in 2013 with the aim of intertwining the work of intersectoral and multidisciplinary organisations with a direct and indirect role in walking in Ireland at national level. The work of the organisation was guided by a national level action plan, the Get Ireland Walking Strategy and Action Plan 2017–2020 (GIW SAP) (19), following its publication in 2017. Gaining an understanding of how the work of Get Ireland Walking aligns with local level walking policies, national level targets, and the global agenda would benefit the next iteration of the GIW SAP. As of October 2022, Get Ireland Walking were undergoing the development stages of a new national walking strategy—succeeding the organisations' previous document—which will be published in 2023.

This aim of this paper is to provide a critical overview of walking policy at local and national level in Ireland across multiple domains using multiple methods with the intention of informing the next iteration of national walking policy in Ireland.

## Methods and materials

This paper uses a mixed methods approach to analyse local and national level walking policies in Ireland across multiple domains. Firstly, a content analysis of local and national walking policies in Ireland was conducted. Secondly, conceptual linkage exercises were carried out to identify the potential contribution of walking, and the specific work of GIW, in attaining national and global level goals. The methods utilised for both objectives are described separately below.

### Objective 1: conduct a content analysis of national and local level walking policies in Ireland

**Figure 1:**
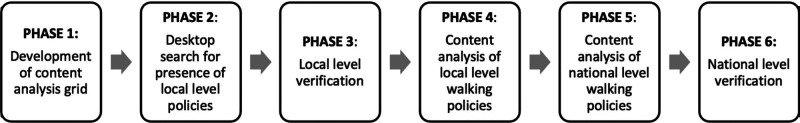
Workflow for local and national level walking policy analysis.

A content analysis of local and national level walking policies in Ireland was conducted using a multi-phased approach. As outlined in Figure 1, there were six phases involved in the content analysis of walking related policies in Ireland. The methods utilised in each phase is described below.

#### Phase 1: development of content analysis grid

To assess local and national level walking policy documents, a content analysis grid was developed and adapted from two existing PA policy audit/assessment tools. The two tools were: (1) The HARDWIRED criteria (20); and (2) the Comprehensive Analysis of Policy on Physical Activity (CAPPA) framework (21). The purpose of the development of a content analysis grid was to ensure a standardised process of assessing the quality of PA policies according to a set of indicators (22).

The CAPPA framework provides a conceptual framework within which to frame analyses of PA policy and was developed through an extensive review of literature, an open discussion between authors, a multiple phase Delphi process and a consultation process with PA policy stakeholders (21). The CAPPA framework allows researchers to situate and direct the scope of research studies relating to the assessment and auditing of PA policies across six categories: (1) Purpose of analysis; (2) Policy level; (3) Policy sector; (4) Type of policy; (5) Stage of policy cycle; and (6) Scope of the analysis. The “Scope of the Analysis” section outlines over twenty sample questions which users of the CAPPA framework can utilise to guide the analysis of PA policy across seven areas (Availability; Context; Processes; Actors; Political Will; Content; and Effects).

The HARDWIRED criteria are a set of characteristics of national PA-related policy which are deemed “absolutely essential” in order for PA policies to achieve successful outcomes at the population level (20). The methodology used by Bellew and colleagues (20) to develop the list of criteria comprised of a literature and policy review, audit of relevant websites, document searches and surveys of international stakeholders. The criteria are: (1) Highly consultative in development; (2) Active through multi-strategic, multi-level, partnerships; (3) Resourced adequately; (4) Developed in stand-alone and synergistic policy modes; (5) Widely communicated; (6) Independently evaluated; (7) Role-clarified and performance delineated; (8) Evidence-informed and Evidence-generating; and, (9) Defined national guidelines for health enhancing physical activity. Short statements are provided for each criterion, allowing users of the HARDWIRED criteria to rate PA policies of interest in accordance with the extent they meet the criteria.

To combine both tools, questions were formulated by the lead researcher (DP) which represented the short statements outlined within each of the HARDWIRED criteria and combined with the corresponding heading of the Scope of the Analysis section in the CAPPA framework (Supplementary file S1). Following this process, a combined list of questions (*n* = 36) across the seven headings in the scope of the analysis section of the CAPPA framework (21) was developed. Several questions deemed to be eliciting similar information were removed and a final composite content analysis tool containing twenty-three (*n* = 23) questions was used to assess local and national level walking policies in Ireland (Supplementary file S1). The approach taken to PA policy content analysis replicates the process used by Daugberg and colleagues (22), who used a content analysis grid across a range of indicators to analyse the contents of 27 national PA policies in the European region.

#### Phase 2: desktop search for presence of local walking polices

Formative research was conducted to provide the contextual backdrop to local and national walking policies in Ireland. Firstly, online searches of local authority websites and grey literature were conducted which aimed to investigate the presence of local level walking policies for all counties within the Republic of Ireland (*n* = 26). Local Authority, Local Sports Partnership, and other relevant websites were searched manually for policy documents relating to the promotion and development of walking. Formal written policies, as per the definition offered by Klepac-Pogrmilovic et al. (21), which focused specifically on the promotion and development of walking or included walking as part of an active travel or walking and cycling related strategy, were included for analysis. County Development Plans (CDP) and Local Sports Partnership Strategic Plans were not included for analysis as not all counties had published a CDP at the time of analysis and is beyond the scope of the current study.

#### Phase 3: local level verification phase

An employee of all Local Sports Partnerships (*n* = 29) was purposively recruited (*n* = 18, 69% response rate) for a short follow up phone call. The purpose of the phone calls was to clarify the findings of the desktop research (Phase 2). Contact details were obtained from the openly accessible Sport Ireland directory of Local Sports Partnership contact details on the Sport Ireland website. Findings relating to the presence of walking related policies in each county were separated into three categories: (a) No walking policy document; (b) Outdated walking policy; and (c) Walking policy present (2015-present). All policy documents retrieved from the online search which met the inclusion criteria and were in the implementation phase no earlier than 2015 were included for further analysis using the adapted content analysis grid developed in Phase 1. All policies found to meet these criteria but preceded 2015 were labelled as “outdated” and not included for further analysis. Policies older than 2015 were excluded due to changes in many contextual factors including COVID-19.

#### Phase 4: content analysis of local level walking policies

The content of each local level walking policy was investigated by the lead researcher (DP) through the application of the content analysis grid. The use of a content analysis grid allows researchers to identify differences among documents according to a list of criteria. The lead researcher (DP) screened each local level walking policy and provided statements for each of the criterion (*n* = 23) outlined within the content analysis grid. The accuracy of the statements provided for each policy were clarified by the authorship team (NM & BL).

#### Phase 5: content analysis of national level walking specific policies

Given the embedded role of the lead researcher (DP) within Get Ireland Walking and the academic and practical experience of the authorship team (BL & NM) in the areas of PA promotion and policy development in Ireland, there was a pre-existing knowledge base in relation to national level walking specific policies in Ireland. Get Ireland Walking's first strategic document, the GIW SAP (19), was published in 2017 and was the only national level walking specific policy document at the time of writing. The GIW SAP outlined 41 actions to be delivered across seven thematic areas by 30 multidisciplinary organisational partners.

#### Phase 6: national level verification

Following the process outlined in Phase 5, a senior member of the GIW staff provided additional details and substantiated the findings in an online meeting which was convened between the lead researcher (DP) and the programme manager of GIW. Specific clarification was sought on questions relating to context (Question 4), processes (Questions 6 and 7), actors (Questions 9 and 11), and political will (Question 12) (Supplementary File S1).

### Objective 2: assess the contribution of (a) walking, and (b) Get Ireland Walking Strategy and Action Plan 2017–2020, to attaining national and global level targets

**Figure 2:**
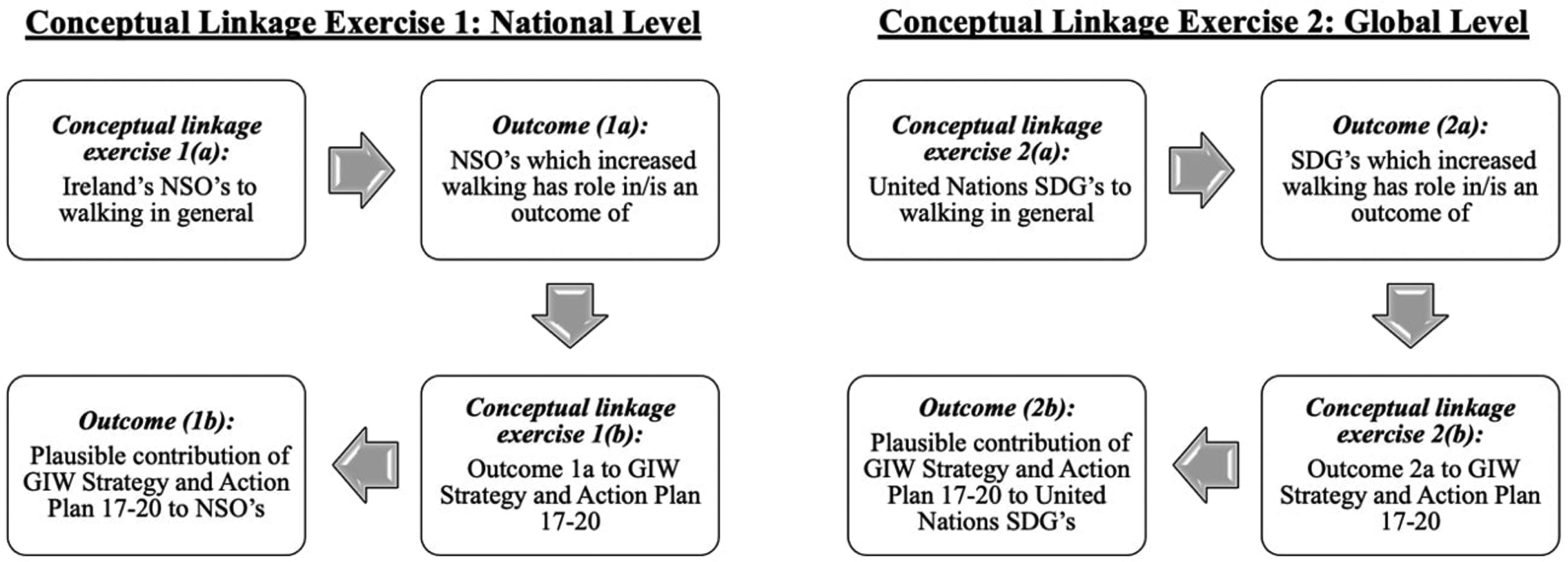
Conceptual linkage exercises workflow. (NSOs, National Strategic Outcomes; SDG's, Sustainable Development Goals).

Conceptual linkage exercises were conducted to understand the contribution of walking, and the GIW SAP, to attaining Ireland's national targets and global level targets set by the United Nations. Figure 2 outlines the workflow involved in the completion of all conceptual linkage exercises conducted as part of this study. The Government of Ireland published the National Development Plan 2021–2030 which outlines ten National Strategic Outcomes (NSOs) for the Irish government to achieve over a ten year period in relation to health, transport, education, and climate change (23). At the global level, the 2030 Agenda for Sustainable Development published by the United Nations outlines 17 goals, termed the Sustainable Development Goals (SDGs), which require international, national, and local partnerships to achieve high-level goals which aim to improve health, education, reduce inequalities, tackle climate change and end poverty across all United Nations member states (5).

Conceptual linkage exercises were conducted to assess how the GIW SAP may contribute to attaining national (NSOs) and global (SDGs) targets which were identified as relevant to walking. All the conceptual linkage exercises followed a similar process to that described by Salvo et al. (6), which relied on deductive logic and the expertise of researchers.

#### Conceptual linkage exercises 1(a) and 2(a): the contribution of walking to national and global goals

Members of the authorship team (DP & NM) are involved in forthcoming work from inFocus Consulting and Sport Ireland which identified 11 SDGs and 47 SDG targets that were related to PA, physical education, and sport policy in Ireland. These findings were used as the basis of the current study. Therefore, at the global level, 47 SDG targets from 11 SDGs were screened and rated in accordance to their relevance to walking. At the national level, 89 targets from 10 NSOs were screened and rated in accordance to their relevance to walking. Walking, in this context, means “more people walking more often” and can hold a bidirectional relationship with SDG targets. For example, SDG Target 3.4 “By 2030, reduce by one third premature mortality from non-communicable diseases through prevention and treatment and promote mental health and well-being” can be partly achieved through increased levels of PA which can be partly obtained by increases in walking levels at the population level. In another example, the SDG Target 11.7 “By 2030, provide universal access to safe, inclusive and accessible, green and public spaces, in particular for women and children, older persons and persons with disabilities” can offer spaces and places for people to walk more often. The lead researcher (DP) conducted the initial screening and rated each SDG and NSO target in accordance to their relevance to walking (highly relevant; partially relevant; not relevant). This rating was subjective and relied on the knowledge of the researcher and their practical experience of being embedded in a national walking promotion organisation. The accuracy of the ratings assigned to all NSO and SDG targets by the lead researcher (DP) was confirmed by the authorship team (NM & BL) and disagreements were resolved through critical discussion.

#### Conceptual linkages exercise 1(b) and 2(b): the contribution of the Get Ireland Walking Strategy and Action Plan 2017–2020 to national and global level targets

A similar exercise was carried out to highlight the contribution of specific actions within the GIW SAP (*n* = 41) to global (SDG) and national (NSO) goals. Only SDG and NSO targets identified as highly relevant and partially relevant to walking in conceptual linkage exercises 1(a) and 2(a) were included for further analysis exercises 2(a) and 2(b), respectively. The United Nations SDG targets and the GIW SAP actions were linked if the successful implementation of the Get Ireland Walking action, at scale, was identified by the lead author (DP) and authorship team (NM & BL) to have the potential to contribute to attaining an SDG target. For example, SDG target 3.4 “By 2030, reduce by one third premature mortality from non-communicable diseases through prevention and treatment and promote mental health and well-being” can partly be achieved through increasing PA levels in those which are the most inactive. Get Ireland Walking implement community-based walking programmes nationally which target inactive population groups, and is an action outlined in the GIW SAP (Action 5.1) (19). The lead researcher (DP) in the current example, identified a plausible contribution of the successful implementation of Action 5.1 in the GIW SAP and the attainment of SDG target 3.4. This process was replicated for all SDG and NSO targets. The authorship team (NM & BL) confirmed the accuracy of the initial ratings of the lead researcher (DP) and disagreements were resolved through critical discussion.

## Results

### Objective 1: conduct a content analysis of national and local level walking strategies in Ireland

#### Local level walking policies in Ireland

**Figure 3:**
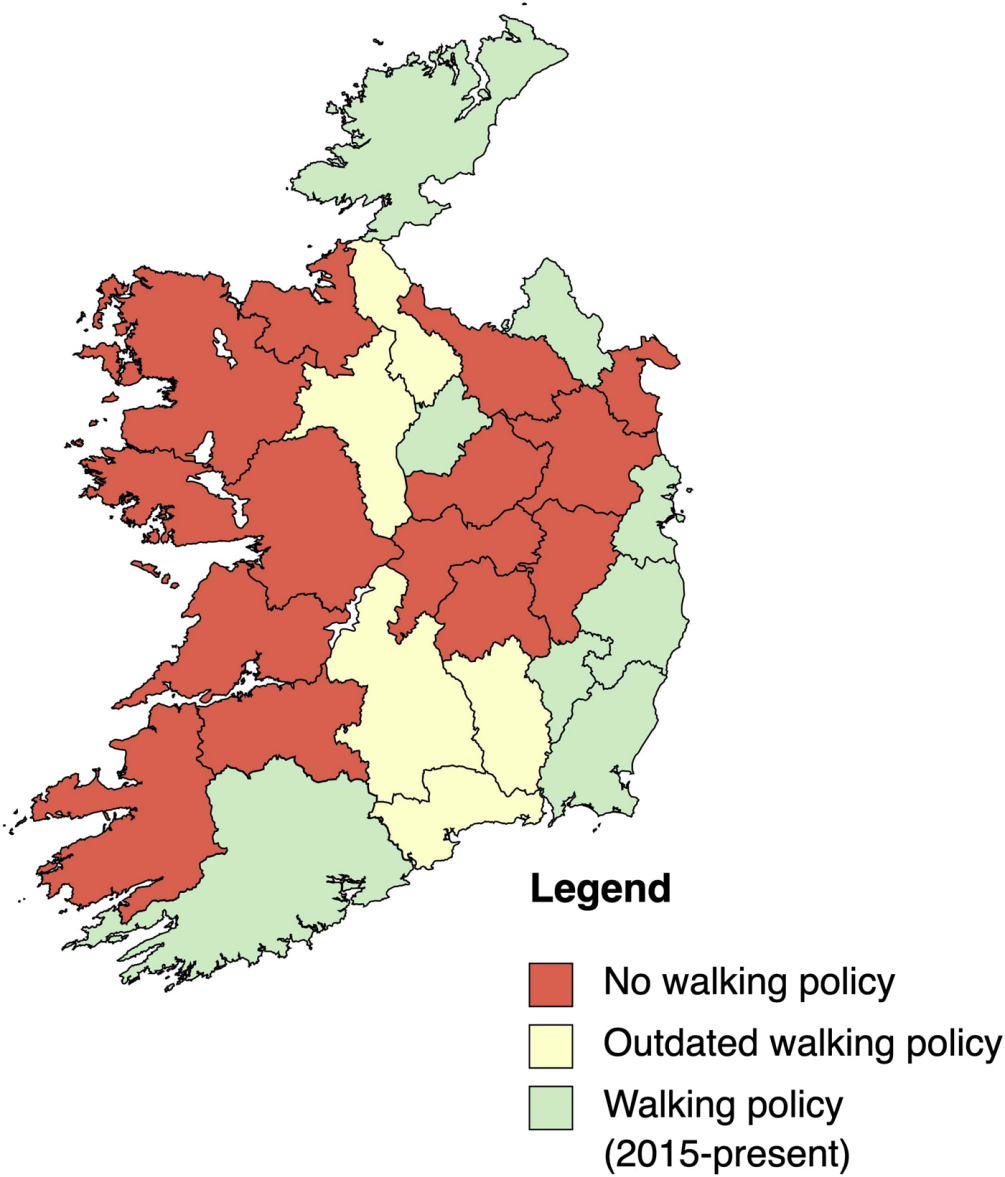
County by county breakdown of presence of walking policies. (Credit: mapchart.net).

Overall, the findings from this study suggest that half (*n* = 13) of counties in the Republic of Ireland do not currently, or have never had, a walking related local level policy document. Figure 3 provides a map of the counties within the Republic of Ireland according to the presence of local level walking policies. For the counties that were found to have a local level walking policy in the implementation phase between 2015 and the time of writing (*n* = 8) (24–31), only one county (Cork) (24) was found to have a walking specific policy. The remaining policies contained walking related actions within a broader scope, including walking and cycling (*n* = 1) (29), tourism (*n* = 2) (28, 30), outdoor recreation (*n* = 2) (25, 31), urban design (*n* = 1) (27), and greenway development (*n* = 1) (26). Five counties were found to have outdated walking related policies. Of these, Waterford was the only county identified to have had a walking specific policy which, at the time of writing, was outdated by almost two decades (32). Below is a summary of results from the application of the content analysis grid to each walking related policy from 2015-present (*n* = 8). Full details of the content analysis can be found in Supplementary file S2.

##### Context

All local level walking related policies included in the content analysis (*n* = 8) were found to have outlined the broader policy context within which the policy sits. Furthermore, all policies outlined the relationship of the policy to other local and national policies from multiple sectors including health, planning, transport, and tourism. However, walking promotion and development was not the primary objective in all policies. One county (Wicklow) (31) specified the nature of funding sources supporting the implementation of the policy, whereas the funding sources supporting the implementation of policies in all other counties was unknown.

##### Processes

All policies included in the content analysis were consultative in development. The processes involved in policy development in all eight counties involved activities such as public meetings, interviews and online questionnaires. Less than half (*n* = 3) of local level policies included in the analysis conducted a context specific needs assessment to direct the actions within the policies (28–30). The majority (*n* = 5) (24, 25, 28–31) of policies included actions relating to the development of a communications strategy to support the implementation of the policy.

##### Actors

All (*n* = 8) policies were multidisciplinary in nature. Organisations from multiple sectors including health, outdoor recreation, sport, local government and tourism were engaged in the development processes of all policies included in the analysis.

##### Political will

There was no information relating to sustained political and stakeholder support on an ongoing basis or in the development process of any policy included in the analysis.

##### Content

Seven of the eight policies outlined the identified timelines for the implementation period of the policy (24, 25, 27–31). One policy (Donegal) (26) specified no timeframe for implementation. Half (*n* = 4) (25, 28, 30, 31) of the policies included a combination of upstream and downstream actions, one policy contained predominantly downstream actions (29), two (26, 27) contained predominantly upstream actions, and one was unclear (24).

##### Effect

The evaluation and monitoring mechanisms included in most policies was poor. Over half (*n* = 5) (21, 24, 26, 27, 30) of the policies did not specify any mechanisms to evaluate the implementation of the policy. For those that did (*n* = 3), two were found to include internal monitoring mechanisms, i.e., stakeholders self-report implementation progress (28, 29) and one highlighted a local university as a body that would assist with evaluation (25).

#### National level walking policies in Ireland

The following sections provides an overview of the application of the composite policy content analysis tool to the only walking-specific national level strategic document in Ireland, the GIW SAP (19) (see Table 1).

**Table 1:** Content analysis of the Get Ireland Walking Strategy and Action Plan 2017–2020.

Scope of policy analysis section	Composite policy audit checklist	Get Ireland Walking Strategy and Action Plan (2017–2020)
Availability	1. Is there a national walking strategy for Ireland?	Yes.
Context	2. What was the key stimulus for policy action?	The previous year (2016) saw the publication of Ireland's first National Physical Activity Plan, within which Get Ireland Walking were a key partner on delivering Action 43 aimed to increase the number of community walking programmes across the Local Sports Partnership network by 100 per annum.
	3. Were local level strategies developed according to the separation of powers doctrine?	No. However, there is work ongoing in Cork as part of a PhD project which will feed into the next iteration of the national strategy.
	4. What budget was allocated for the implementation of the policy?	No specific budget for the implementation of Strategy. In 2017, the initiative received €145k from Sport Ireland and Healthy Ireland. The Get Ireland Walking initiative was awarded an additional €100k through Dormant Accounts funding in 2018.
	5. Does the policy have a clear statement which is also embedded in other policy agendas?	The vision of Get Ireland Walking is to maximise the amount of people who walk regularly on the island of Ireland. Smarter Travel and the Design Manual for Urban Roads and Streets are both related policy documents yet outline a broader agenda.
Processes	6. What process did the strategy have to go through to be implemented?	Advisory group drafted initial draft of the Strategy. Individual interviews with stakeholders (*n* = 30) from government agencies, sporting bodies, charities, and not-for-profit were conducted to determine actions they could they lead on and could collaborate on. Stakeholder decided whether they could lead or collaborate on specific actions.
	7. Was a stakeholder analysis and needs assessment conducted to ensure widespread representation from interdisciplinary stakeholders at the early stages of strategy development?	Members of the advisory group (*n* = 15) (chaired by Sport Ireland) nominated relevant stakeholders to engage in the strategy development process. No reference to stakeholder analysis or needs assessment.
	8. What mechanisms are in place to support the dissemination of the strategy?	Action 1.1: Develop and implement a three-year Get Ireland Walking communications strategy. Get Ireland Walking communications strategy was published in 2019. No document outlining tailoring of Strategy content to needs of heterogenous stakeholders i.e., policymakers, researchers.
Actors	9. Does the strategy engage with grassroots practitioners, as well as policymakers, and define the organisational links between them?	30 partners organisations from multiple sectors mentioned as key partners and/or collaborators in the strategy. Organisations operate at levels ranging from policymakers to local level practitioners.
	10. What were the power relations between the actors involved in the development process?	Organisations involved at the consultation process, although have local level work programmes, all operate nationally. Organisations such as Department of Health, Health Service Executive and Sport Ireland are key policymaking organisations and provide core funding to other organisations on the list of partners and collaborators. For example, Local Sports Partnerships funded by Sport Ireland, Irish Heart Foundation part-funded through Health Service Executive.
	11. Were actions within the strategy progressed through intersectoral partnerships?	Yes, as most actions within the strategy were the responsibility of organisations from multiple sectors. However, no insight into the extent to which actions were implemented or evaluated. Progressed monitored only through self-report traffic light system (annually).
Political will	12. Did any political actor in power publicly express support to the development of the strategy?	An Taoiseach Leo Varadkar and Minister for Health Minister Simon Harris officially launched the Strategy in 2017.
	13. Is there a stable base of political and stakeholder support as well as sustained investment over the long term?	The Get Ireland Walking initiative is funded through dormant accounts funding, Healthy Ireland and Sport Ireland funding streams and is reviewed on an annual basis.
	14. Does the government hold regular discussions with the aim to support the implementation of the strategy?	No.
Content	15. Are the roles and responsibilities of organisations involved in strategy implementation well clarified and is there a common understanding of and agreement on how “successful implementation” is to be defined and measured?	Organisations have been assigned as either (a) lead partners of (b) collaborators on all actions within the Strategy. No consensus on successful implementation, evaluation, or dates for accountability purposes outlined. Progressed monitored only through self-report traffic light system (annually).
	16. Does the strategy have a clear statement on the timeframe for policy implementation?	Yes (2017–2020). Annual deadlines assigned to the implementation of specific actions. Get Ireland Walking implements an operational plan internally with the support and guidance of the National Governing Body, Mountaineering Ireland.
	17. Does the strategy reference specific target groups?	Yes. Actions within various themes focus on children and young people, mental health service users, and community-based walking programmes for inactive populations. Lead organisations are assigned to each action.
	18. Is the policy content predominantly “downstream” or “upstream”?	Combination of both. There are both examples of actions which pertain to the implementation of community-based programmes (downstream) and facilitating policy alignment across sectors (upstream) mentioned within the strategy.
	19. Does the strategy outline a comprehensive approach using multiple strategies at multiple levels targeting multiple population groups?	Yes. The Strategy outlines actions which range from individual level interventions to higher level interventions.
Effects	20. Is the evaluation conducted by an independent body which is not connected to the government or “policy owners”?	The overall Get Ireland Walking initiative was evaluated by a consultancy company in 2022. The evaluation involved the co-development of key performance indicators and evaluated Get Ireland Walking on progress to those key performance indicators since 2013. Self-report traffic light system was in place throughout the implementation of the Strategy results are unknown.
	21. Is there systematic surveillance of population levels of walking?	Yes. The Irish Sports Monitor monitors trends in self-reported recreational and transport walking data biannually in Ireland. Transport related walking monitored in Census every five years.
	22. What kind of impact did the strategy have on walking levels?	Unknown/Not measured.
	23. Were there any unintended consequences of the implementation of the strategy?	Unknown/Not measured.

##### Context

Get Ireland Walking received funding from Sport Ireland and Healthy Ireland 2017. In 2018, annual funding for the initiative increased through the Dormant Account funds. The GIW SAP, at the time of publication, sat within the broader national PA policy context in Ireland. For example, Action 43 of the National Physical Activity Plan 2016–2020 (33) outlines Get Ireland Walking as a lead partner. Although Get Ireland Walking has both national and local remits, the GIW SAP was found to lack local level delivery mechanisms which feed into the implementation of the GIW SAP at national level. Given the lack of political leverage of Get Ireland Walking, there was little capacity to embed actions into interagency programmes of work to ensure accountability and transparency.

##### Processes

In order to progress the GIW SAP to the implementation phase, the Get Ireland Walking advisory group, consisting of 15 stakeholders from Sport Ireland, the Department of Health, Get Ireland Walking, the Health Service Executive, Ireland Active, the Irish Heart Foundation, and Mountaineering Ireland, developed the preliminary list of actions and nominated organisations to implement the actions as lead organisations or collaborators. Following this, a consultation process of 30 individual interviews with partner organisations were conducted in 2016 to determine the capacity for nominated organisations to act as lead partners or collaborators on assigned actions.

##### Actors

Actions within the GIW SAP were assigned to intersectoral organisations operating at multiple levels. For example, the GIW SAP engages with organisations operating at grass roots level (i.e., Local Sports Partnership network) and policymakers (i.e., Department of Health). Progress relating to the implementation of the actions within the GIW SAP were monitored through an annual self-report monitoring report completed by organisations. This was completed inconsistently over the implementation period of the GIW SAP.

##### Political will

Throughout the development process of the GIW SAP, no government official or political figure supported or engaged in the development process of the GIW SAP. However, the GIW SAP was officially launched by An Taoiseach (Prime Minister) Leo Varadkar and Minister for Health Simon Harris in 2017.

##### Content

The overall implementation period of the GIW SAP is clearly defined (2017–2020) and annual timelines are assigned to each action (i.e., completed by end of 2019). The content of the actions and thematic areas outlined within the GIW SAP varies and outlines actions and sections which focus on specific target groups (i.e., children and young people). Although lead partners and collaborators are assigned to each action, the exact roles of each organisation and what represents successful implementation is not stated.

##### Effects

The GIW SAP was not evaluated independently. However Get Ireland Walking (as an initiative of Sport Ireland) was independently evaluated in 2022. Progress on the implementation of the GIW SAP was monitored annually through stakeholders self-reporting their progress on actions according to a traffic light system. Although the Irish Sports Monitor is an established national level survey measuring self-reported recreation and transport walking, the impact of the SAP on population levels of walking in Ireland is unknown.

### Objective 2: assess the contribution of (a) walking, and (b) Get Ireland Walking Strategy and Action Plan 2017–2020, to attaining national and global level targets

The results for Objective 2 are presented in four sections. Each section relates to the outcomes of the four conceptual linkage exercises outlined in Figure 2 to assess the contribution of walking to attaining global and national level targets (1a and 2a), and the contribution of the GIW SAP to attaining global and national level targets (1b and 2b).

#### Conceptual linkage exercise 1(a): the contribution of walking in attaining national level targets in Ireland

Overall, there were 88 NSO targets across 10 NSOs which were screened by the authorship team. Following the conceptual linkage exercise, 28 NSO targets within six NSOs were identified to hold bi-directional relationships to walking. Specific target statements were identified within NSOs which were related to sustainable mobility (NSO 4), strengthening local economy (NSO 3), improving access to amenities (NSO 7), quality education and healthcare (NSO 10), intercity accessibility (NSO 2) and sustainable growth of towns and cities (NSO 1). Table 2 highlights the full list of NSO targets which were identified as partially or highly relevant to walking. Of the 28 NSO target statements identified as relevant to walking, over half (*n* = 15) of the targets were identified as highly relevant to walking.

**Table 2:** National strategic outcomes (NSO) and associated NSO targets identified as relevant to walking.

National Strategic Outcome	National Strategic Outcome Target Statement	NSO target relevance to walking
NSO 1: Compact Growth	1.1 - Enable urban infill development that would not otherwise occur	Partially relevant
	1.2 - Improve “liveability” and quality of life, enabling greater densities of development to be achieved	Highly relevant
	1.3 - Encourage economic development and job creation, by creating conditions to attract internationally mobile investment and opportunities for indigenous enterprise growth	Partially relevant
	1.4 - Building on existing assets and capacity to create critical mass and scale for regional growth	Partially relevant
	1.5 - Improve accessibility to and between centres of mass and scale and better integration with their surrounding areas	Highly relevant
	1.6 - Ensure transition to more sustainable modes of travel (walking, cycling, public transport) and energy consumption (efficiency, renewables) within an urban context	Highly relevant
	1.7 - Encourage labour mobility to support employment-led growth, including affordable housing, education/skills development and improved community and family services including childcare	Partially relevant
	1.8 -Enhance the attractiveness, viability and vibrancy of smaller towns and villages and rural areas as a means of achieving more sustainable patterns and forms of development	Highly relevant
	1.9 - Ensure transition to more sustainable modes of travel (walking, cycling, public transport) and energy consumption (efficiency, renewables) within smaller towns and villages and rural areas	Highly relevant
	1.12 - Cross-boundary collaboration at county and regional level to achieve more sustainable outcomes for rural communities, e.g. applicable to shared settlements, landscapes and amenities as well as lands in state ownership	Partially relevant
NSO 2: Enhanced Regional Accessibility	2.3- Enabling more effective traffic management within and around cities and re-allocation of inner city road-space in favour of bus-based public transport services and walking/cycling facilities	Highly relevant
	2.8 - To strengthen public transport connectivity between cities and large growth towns in Ireland and Northern Ireland with improved services and reliable journey times	Partially relevant
NSO 3: Strengthened Rural Economies and Communities	3.1 - Implementation of the actions outlined in the Action Plan for Rural Development	Partially relevant
	3.3 - Implementation of a targeted Rural Regeneration and Development Fund to enable opportunities to secure the rejuvenation and re-purposing of rural towns and villages weakened by the structural changes in rural economies and settlement patterns	Partially relevant
	3.4 - Provide a quality nationwide community based public transport system in rural Ireland which responds to local needs under the Rural Transport Network and similar initiatives	Partially relevant
	3.5 – Invest in maintaining regional and local roads and strategic road improvement projects in rural areas to ensure access to critical services such as education, healthcare and employment	Partially relevant
	3.6 - Invest in greenways, blueways and peatways as part of a nationally coordinated strategy	Highly relevant
NSO 4: Sustainable mobility	4.1 - Expand attractive public transport alternatives to car transport to reduce congestion and emissions and enable the transport sector to cater for the demands associated with longer-term population and employment growth in a sustainable manner through the following measures	Highly relevant
	4.2 - Deliver the key public transport objectives of the Transport Strategy for the Greater Dublin Area 2016–2035 by investing in projects such as New Metro Link, DART Expansion Programme, BusConnects in Dublin and key bus-based projects in the other cities and towns	Partially relevant
	4.3 - Provide public transport infrastructure and services to meet the needs of smaller towns, villages and rural areas	Partially relevant
	4.4 - Develop a comprehensive network of safe cycling routes in metropolitan areas to address travel needs and to provide similar facilities in towns and villages where appropriate	Partially relevant
NSO 7: Enhanced Amenities and Heritage	7.1 - Implementation of planning and transport strategies for the five cities and other urban areas will be progressed with a major focus on improving walking and cycling routes, including continuous greenway networks and targeted measures to enhance permeability and connectivity	Highly relevant
	7.2 - The Rural and Urban Regeneration and Development Funds will support transformational public realm initiatives to give city and town centre areas back to citizens, encouraging greater city and town centre living, enhanced recreational spaces and attractiveness from a cultural, tourism and promotional perspective	Highly relevant
	7.3 - We will conserve, manage and present our heritage for its intrinsic value and as a support to economic renewal and sustainable employment	Partially relevant
	7.4 - Open up our heritage estates to public access, where possible	Highly relevant
	7.5 - Invest in and enable access to recreational facilities, including trails networks, designed and delivered with a strong emphasis on conservation, allowing the protection and preservation of our most fragile environments and providing a wellbeing benefit for all	Highly relevant
NSO 10: Access to Quality Childcare, Education and Health Services	10.1 - Provide additional investment in the schools sector to keep pace with demographic demand and to manage increasing building and site costs so that new and refurbished schools on well-located sites within or close to existing built-up areas, can meet demographic growth and the diverse needs of local population	Highly relevant
	10.2 - Expand and consolidate third-level facilities at locations where this will further strengthen the capacity of those institutions to deliver the talent necessary to drive economic and social development in the regions. The consolidation of the DIT campus at Grange Gorman is a critical flagship infrastructural project for the higher education sector	Partially relevant

#### Conceptual linkage exercise 2(a): the contribution of walking in attaining global level targets

Forty-seven (*n* = 47) SDG targets from 11 SDGs were screened in accordance to their relevance to walking by the authorship team. Overall, there were 8 SDGs (SDG 3; SDG 4; SDG 8; SDG 11; SDG 12; SDG 13; SDG 16; and SDG 17) which were identified as relevant to walking. More specifically, 19 SDG targets across the 8 SDG's were found to be highly relevant (*n* = 8, 42%) or partially relevant (*n* = 11, 58%) to walking. The full list of SDG targets and their relevance to walking can be found in Table 3.

**Table 3:** Sustainable development goals and associated SDG targets identified as relevant to walking.

SDG	SDG target	SDG target relevance to walking
SDG 3: Good health and well-being	3.4: By 2030, reduce by one third premature mortality from non-communicable diseases through prevention and treatment and promote mental health and well-being	Highly relevant
	3.5: Strengthen the prevention and treatment of substance abuse, including narcotic drug abuse and harmful use of alcohol	Partially relevant
	3.6: By 2020, halve the number of global deaths and injuries from road traffic accidents	Highly relevant
	3.9: By 2030, substantially reduce the number of deaths and illnesses from hazardous chemicals and air, water and soil pollution and contamination	Partially relevant
SDG 11: Sustainable cities and communities	11.2: By 2030, provide access to safe, affordable, accessible and sustainable transport systems for all, improving road safety, notably by expanding public transport, with special attention to the needs of those in vulnerable situations, women, children, persons with disabilities and older persons	Highly relevant
	11.3: By 2030, enhance inclusive and sustainable urbanisation and capacity for participatory, integrated and sustainable human settlement planning and management in all countries	Highly relevant
	11.6: By 2030, reduce the adverse per capita environmental impact of cities, including by paying special attention to air quality and municipal and other waste management	Highly relevant
	11.7: By 2030, provide universal access to safe, inclusive and accessible, green and public spaces, in particular for women and children, older persons and persons with disabilities.	Highly relevant
	11.a: Support positive economic, social and environmental links between urban, peri-urban and rural areas by strengthening national and regional development planning.	Highly relevant
SDG 4: Quality Education	4.7: By 2030, ensure that all learners acquire the knowledge and skills needed to promote sustainable development, including, among others, through education for sustainable development and sustainable lifestyles, human rights, gender equality, promotion of a culture of peace and non- violence, global citizenship and appreciation of cultural diversity and of culture's contribution to sustainable development	Partially relevant
SDG 8: Decent work and economic growth	8.1: Sustain per capita economic growth in accordance with national circumstances and, in particular, at least 7 per cent gross domestic product growth per annum in the least developed countries	Partially relevant
	8.9: By 2030, devise and implement policies to promote sustainable tourism that creates jobs and promotes local culture and products	Highly relevant
SDG 12: Responsible consumption and production	12.8: By 2030, ensure that people everywhere have the relevant information and awareness for sustainable development and lifestyles in harmony with nature	Partially relevant
	12.2: By 2030, achieve the sustainable management and efficient use of natural resources	Partially relevant
SDG 13: Climate action	13.2: Integrate climate change measures into national policies, strategies and planning	Partially relevant
SDG 16: Peace, justice and strong institutions	16.6: Develop effective, accountable and transparent institutions at all levels	Partially relevant
	16.7: Ensure responsive, inclusive, participatory and representative decision- making at all levels	Partially relevant
SDG 17: Partnerships for the goals	17.16: Enhance the Global Partnership for Sustainable Development, complemented by multi-stakeholder partnerships that mobilise and share knowledge, expertise, technology and financial resources, to support the achievement of the SDGs in all countries, in particular developing countries	Partially relevant
	17.17: Encourage and promote effective public, public– private and civil society partnerships, building on the experience and resourcing strategies of partnerships	Partially relevant

#### Conceptual linkages exercise 1(b): the contribution of the Get Ireland Walking Strategy and Action Plan 2017–2020 to Ireland's national strategic outcomes

**Figure 4:**
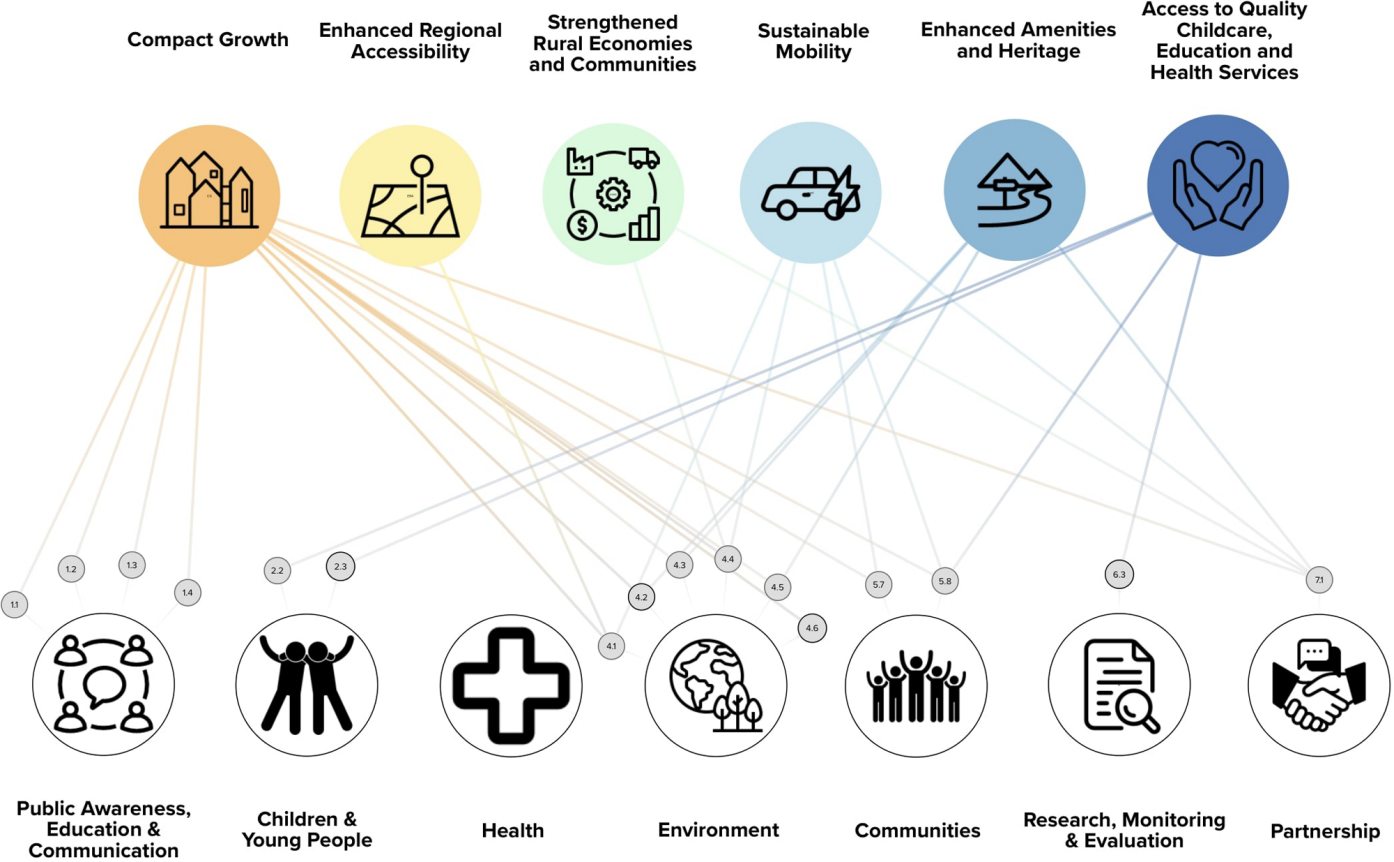
Walking related NSO's and NSO targets (Top row) and their links to actions within the themes of the Get Ireland Walking Strategy and Action Plan 2017–2020 (bottom row) (solid line = NSO target identified as highly relevant to walking; dashed line = NSO target identified as partially relevant to walking.

The findings of the conceptual linkage exercise investigating the contribution of the GIW SAP to Ireland's national level governmental targets (NSOs), suggest that actions in six out seven of the thematic areas listed in the GIW SAP may contribute to six out of ten NSOs. Figure 4 provides an overview of the potential contribution of actions within the GIW SAP to NSO targets. There were a total of 17 GIW SAP actions which were identified to plausibly contribute to attaining 27 NSO targets. The most explicit contributions of actions within the GIW SAP to NSOs were between the Environment theme in the GIW SAP and NSO 1 (Compact Growth) and NSO 7 (Enhanced Amenities and Heritage). Actions within the Environment theme of the GIW SAP were found to hold the potential to contribute to five out of six of the walking related NSOs. There were no actions within the Health theme of the GIW SAP that were identified as contributing to the attainment of any NSO targets. A full list of the GIW SAP actions and their conceptual linkages with NSO targets can be found in Supplementary file S3.

#### Conceptual linkages exercise 2(b): the contribution of the Get Ireland Walking Strategy and Action Plan 2017–2020 to the united nations sustainable development goals

**Figure 5:**
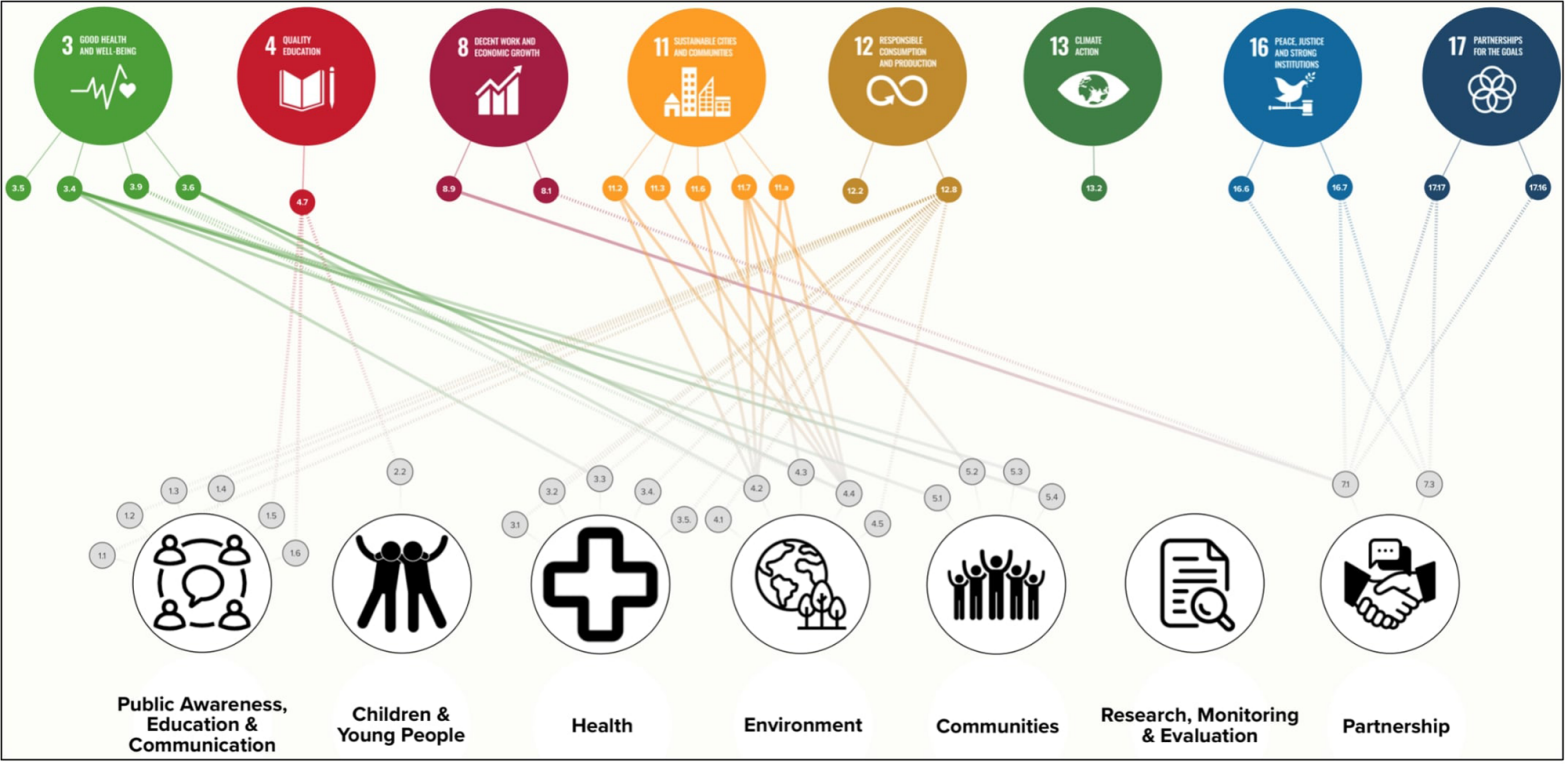
Walking related SDG's and SDG targets (Top row) and their links to actions within the Get Ireland Walking Strategy and Action Plan 2017–2020 (bottom row) (solid line = SDG target identified as highly relevant to walking; dashed line = SDG target identified as partially relevant to walking).

The findings of the conceptual linkage exercise exploring the connection between the GIW SAP and SDG targets suggest that there are ample opportunities to increase the scope of SDG-relevant actions within future iterations of the GIW SAP. For example, the most explicit links between GIW SAP actions and SDG targets were identified between actions in the Environment and Communities themes with SDG 11 (Sustainable Cities and Communities) and SDG 3 (Good Health and Wellbeing), respectively. However, actions across six themes in the GIW SAP were identified as potential contributors to SDG targets across six SDG's. Figure 5 provides a visualisation of the actions within the GIW SAP which could plausibly contribute to the attainment of SDG targets. There was a total of twenty-three (*n* = 23) actions within the SAP which held partial and highly relevant links to sixteen SDG targets. There were three SDG targets (3.5; 12.2; 13.2) which were found to be partially relevant to walking, but not to actions within the GIW SAP. There were no actions within the Research, Monitoring and Evaluation theme of the GIW SAP which were relevant to any walking-related SDG targets. Similarly, there were no GIW SAP actions which held plausible links to the SDG 13 (Climate Action). Sustainable Development Goal 3 (Good health and wellbeing) and SDG 11 (Sustainable Cities and Communities) were found to have the highest number of SDG targets which were identified as highly relevant to actions within the SAP. All walking-related SDG targets in SDG 11 (Sustainable Cities and Communities) were found to hold highly relevant links to three actions in the Environment theme of the SAP, and one action in the Communities theme of the GIW SAP. A full list of the GIW SAP actions and their conceptual linkages with SDG targets can be found in Supplementary file S4.

## Discussion

The aim of this study was to provide a critical overview of walking policy at local and national level in Ireland across multiple domains. The findings from this study are threefold. Firstly, the presence of walking specific local level policies is low. Furthermore, local level walking specific policies in Ireland were found to be vague in nature, lacking clarity on the roles and responsibilities of organisations and information relating to evaluation. Secondly, findings from the content analysis of national level walking policies found that the GIW SAP was, at its core, interdisciplinary in nature yet lacked clarity on the specific roles and responsibilities of organisations involved in the implementation and evaluation of the strategy. Moreover, the GIW SAP holds a national level scope, yet lacks local level delivery mechanisms to assist with the implementation. Thirdly, findings from the conceptual linkage exercises suggest that walking can contribute to many national and global targets, yet there are opportunities to increase the breadth of targets which walking, and the work of Get Ireland Walking, can have through whole-of-systems approaches.

### Irish walking policy—synergies and specificities

Our results suggest that half of the counties in the Republic of Ireland have never had a local level policy with a specific walking focus. For the counties that have, five were dated before 2015, suggesting the need for renewal of some policies. The policies which underwent content analysis as part of the current study were found to be multidisciplinary in nature and were consultative in development. Bellew and colleagues (20) identify multidisciplinary action and consultation as necessities in successful PA related policy. However, ensuring strong monitoring mechanisms are embedded in PA policy is of utmost importance to the overall effectiveness of a policy (34, 35). There were very few examples of effective evaluation and monitoring mechanisms in local and national walking policies identified in the current content analysis. The lack of governance and accountability mechanisms embedded within local walking policies in Ireland found in the current study may be explained by local level walking system actors engaging in symbolic politics, where the development and publication of public policy provides an emblematic gesture to the public, with no real intention of implementation (36–38). However, the transdisciplinary nature of walking may also help explain the lack of governance and monitoring mechanisms in local walking policies in Ireland. Walking promotion and development is not the sole responsibility of one sector, organisation, or discipline. Previous work by our research group (11) has demonstrated the potential for systems science methods, specifically systems mapping, to assist with engaging multidisciplinary stakeholders at local level in Ireland. Although Power and colleagues used systems mapping as a catalyst to engage stakeholders, systems mapping can also help identify data sources and monitoring mechanisms during the stages of developing local walking policies (39).

The results of the content analysis suggest that local level policies included walking as part of broader agendas such as walking and cycling, tourism, and outdoor recreation. Cork and Waterford were found to be the only two counties in the Republic of Ireland which had published walking specific local level policies. Interestingly, both differ in their overall focus. The Cork City Walking Strategy 2013–2018 (24) focuses on the promotion of walking for transport, whereas Step by Step: Walking Strategy for Waterford (32) focuses predominantly on recreational walking. While there are examples of transport specific walking policies in Norway (40), and acknowledging walking as its own transport mode when collecting data and devising policies is recommended (41), it must be noted that this approach to local level walking policies may exacerbate disciplinary siloes. A more systems-oriented approach to walking policy has been adopted by Paths for All, a national walking promotion charity in Scotland (17). Adopting a similar approach to walking promotion in Ireland by embedding national level policy in the global agenda with local level implementation supports may be a positive step for walking promotion in Ireland. An organisation such as Get Ireland Walking has the potential to mobilise recent increases in funding allocated to walking in Ireland (18) and to act as a national level facilitator in cultivating a systems approach to walking through engaging organisations from across sectors and disciplines.

Similar to the majority of local level walking policies, the national level GIW SAP is multidisciplinary in nature and consultative in development. Ireland's only national walking specific policy document was developed after a period of consultation with stakeholders from sport, health, education, transport, and academia. Engaging with multiple sectors has been noted as best practice in the PA policy development literature (20, 21, 42). However, a study conducted by Power et al. (11) which used social network analysis methods to evaluate the communication network between the multidisciplinary actors involved in the implementation of the GIW SAP, found that there was a mismatch between how actors were required to communicate (based on collaborative actions in the GIW SAP) compared with how actors communicated in practice. The lack of clarity in relation to the roles and responsibilities of the stakeholders involved in the implementation of the GIW SAP may be explained by the lack of political leverage held by GIW. For example, GIW does not operate at a governmental level and has limited resources and thus does not hold the capacity to embed the GIW SAP actions within the work of collaborating organisations. For future iterations of national walking policy in Ireland, care should be taken to develop a common vision to ensure effective coordination for policy implementation (43, 44). More research is needed to understand potential for systems-oriented methods such as systems mapping and social network analysis to be embedded within PA policy evaluation plans in conjunction with more traditional methods. Doing so may help stakeholders to develop a common understanding of the policy system and to gain real time insights into the collaboration networks involved in policy implementation.

### How can walking contribute to the attainment of national and global level goals?

Conceptual linkage exercises to explore the contribution of PA and sport to the United Nations SDGs have been conducted elsewhere (6, 13, 14) and the potential benefit to aligning the PA field to the SDGs has been outlined (7). The findings presented in this study build on the approaches used by Salvo et al. (6) and are applied specifically to walking. Using global and national goals as conceptual frameworks within which to view national walking policy in Ireland facilitated the identification of opportunities for deepening the potential contribution that walking can have attaining higher-level targets. However, the contribution of the GIW SAP to national level targets in Ireland is limited. For example, the most explicit contributions of the GIW SAP to the NSOs identified were from actions within the Environments and Communities themes. It has been suggested that PA is perceived to be the sole responsibility of organisations within the health, transport, and sport sectors, when in reality, there are a plethora of sectors who have a role to play (45, 46). The policy actions included in the Environments and Communities themes in the GIW SAP can be interpreted as playing a direct and explicit role in the promotion and development of walking, for example through the implementation of community-based walking programmes (19). However, there is potential to include a wider breadth of future GIW SAP actions that play an indirect role in attaining national level targets in Ireland with sufficient collaboration, coordination and alignment between stakeholders in the walking system.

The findings of the current study indicate that walking can possibly contribute to over half of Ireland's NSO's with ties to many sectors including urban design, planning, local government and transport. However, the contribution of GIW SAP actions were more explicitly relevant to the tourism, health, sport, research, and outdoor recreation sectors. Yet interestingly, many of the targets outlined within each of the NSOs do not explicitly mention walking, pedestrians, or PA—yet were still identified as partially or highly relevant to walking. The lack of breadth in terms of to the actions within the GIW SAP which were identified to potentially contribute to national targets may be partly explained by the context within which GIW is situated. The organisation is not an independent body and operates within Mountaineering Ireland, the national governing body for Mountaineering in Ireland, whose agenda predominantly focuses on the use of mountains and trails for recreational walking. The conceptual work of Piggin is mirrored in practice here within the Irish walking system. Piggin (47) advocates for a more holistic definition of PA which could help incorporate a wider breadth of sectors in policy decisions relating to PA. The use of NSOs and SDGs to facilitate a viewpoint of walking through a broader systems-oriented lens may allow for opportunities to identify organisations and decision makers outside of those who are already engaged in walking policy in Ireland to become evident. Doing so may lead to improved future iterations of Irish walking policy which are transparent with national and global targets.

## Strengths and limitations

There are some limitations in this study. Firstly, although the approach taken to the conceptual linkage exercises mirrored the methods of Salvo et al. (6), there were fewer members of the research team for this study. This may have increased the risk of omitting linkages. However, the research team included a researcher embedded in Ireland's national walking promotion organisation (DP) and experienced researchers in PA policy in Ireland (BL & NM). This research provides a platform upon which to build on and confirm or refute the findings of the conceptual linkage exercise, as the identification of indicator datasets to clarify the existence of an empirical link between national walking policy and national and global goals was beyond the scope of this study. The exclusion of Local Sports Partnership strategic plans and County Development Plans from the analysis may have resulted in the omission of some local level walking related policy actions. The local level verification phase of the content analysis (Phase 3), only involved members of the Local Sports Partnerships network. It is plausible that individuals from other sectors may have provided different responses. However, the Local Sports Partnerships network represent a network of stakeholders embedded in local walking systems which provide the largest geographical spread, and thus were identified as the most appropriate contacts for the current study. This study is an example of how PA policy analysis tools can be used to inform the development of more effective walking strategies and policies that are aligned to national and international targets.

## Conclusion

There is a need to update local and national walking policies in Ireland according to best practice criteria from international PA policy to ensure transparency and alignment across policy levels. This paper provides guidance to local and national walking systems in Ireland on (re)writing walking policies which are transparent with national and global agendas. With multidisciplinary action across walking systems, walking can help contribute to many national and global targets. Developing future policies which strengthen existing connections to national and global targets should be prioritised by local and national walking systems in Ireland.

## Data Availability

The original contributions presented in the study are included in the article/**Supplementary Material**, further inquiries can be directed to the corresponding author/s.
