# Supplementary material for: A critical analysis of walking policy in Ireland and its contribution to both national and international development goals
Source: Front Sports Act Living. 2023 Mar 1;5:1125636. doi: 10.3389/fspor.2023.1125636 (PMC10014795; doi:10.3389/fspor.2023.1125636)
Supplement: Supplementary file 1 [file Table1.docx]

| **CAPPA (Scope of Analysis subheading)** | **CAPPA example questions** | **HARDWIRED example questions** | **Composite list questions** |
| --- | --- | --- | --- |
| **Availability:** Analysis of whether a policy exists or not (e.g., the presence of a national PA plan) | Example questions:  -Is there a national walking strategy for Ireland? | Proposed HARDWIRED questions:  N/A | **1.** Is there a national walking strategy for Ireland/[name of county]? |
| **Context:** Analysis of the economic, environmental, legal, political, social, and any other circumstances relevant to a policy or a stage of policy cycle | Example questions (CAPPA):  -What budget was allocated for the implementation of the policy?  -What was the key stimulus for policy action?  -What are the dominant values held by the body endorsing the strategy?  -What influence does private sector have on policy making process?  -Were local level strategies developed according to the separation of powers doctrine? | Proposed HARDWIRED questions:  **D_1_ –** Does the policy have a clear statement which is also embedded in other policy agendas?  **R_1_ –** Is there a stable base of political and stakeholder support as well as sustained investment over the long term? | **2.** What was the key stimulus for policy action?  **3.** Were local level strategies developed according to the separation of powers doctrine?  **4.** What budget was allocated for the implementation of the policy?  **5.** Does the policy have a clear statement which is also embedded in other policy agendas? |
| **Processes:** Analysis of the procedures, mechanisms, and/or actions in a given stage of the policy cycle. | Example questions (CAPPA):  -What process did the strategy have to go through to be implemented?  -What mechanisms are in place to support the dissemination of the strategy?  -Did the development process allow for suggestions and improvements to be made?  -Which mechanisms were in place in the development stage of the strategy? | Proposed HARDWIRED questions:  **H** – Was a stakeholder analysis and needs assessment conducted to ensure widespread representation from interdisciplinary stakeholders at the early stages of strategy development?  **W** – Was communication of the contents of the strategy tailored before disseminating with different target groups? | **6.** What process did the strategy have to go through to be implemented?  **7.** Was a stakeholder analysis and needs assessment conducted to ensure widespread representation from interdisciplinary stakeholders at the early stages of strategy development?  **8.** What mechanisms are in place to support the dissemination of the strategy? |
| **Actors:** Analysis of the stakeholders in a given stage of a policy cycle. | Example questions (CAPPA):  -Who were the bodies involved in the development of the policy?  -Which bodies proposed the strategy?  -What were the power relations between the actors involved in the development process?  -Are any non-governmental organisations assisting in the implementation of the policy? | Proposed HARDWIRED questions:  **H** – Does the strategy engage with grassroots practitioners, as well as policymakers, and define the organisational links between them?  **A** – Were actions within the strategy progressed through intersectoral partnerships? | **9.** Does the strategy engage with grassroots practitioners, as well as policymakers, and define the organisational links between them?  **10.** What were the power relations between the actors involved in the development process?  **11.** Were actions within the strategy progressed through intersectoral partnerships? |
| **Political will:** Analysis of the level of political support and/or commitment to a policy in a given stage of the policy cycle. | Example questions (CAPPA):  -Did any political actor in power publicly express support to the development of the strategy?  -Did the Government demonstrate political will to support the implementation of the strategy?  -Does the government hold regular discussions with the aim to support the implementation of the strategy? | Proposed HARDWIRED questions:  **R_1_** – Is there a stable base of political and stakeholder support as well as sustained investment over the long term? | **12.** Did any political actor in power publicly express support to the development of the strategy?  **13.** Is there a stable base of political and stakeholder support as well as sustained investment over the long term?  **14.** Does the government hold regular discussions with the aim to support the implementation of the strategy? |
| **Content:** Analysis of the wording and substantive information included in a specific policy. | Example questions (CAPPA):  -Does the strategy reference specific target groups?  -Does the strategy have a clear statement on the timeframe for policy implementation?  -Does the strategy mention joint collaboration at different levels of government?  -Are the national PA recommendations in your country fully in line with the WHO recommendations for PAfH?  -Is the policy content predominantly ‘downstream’ or ‘upstream’? | Proposed HARDWIRED questions:  **R_2_ –** Are the roles and responsibilities of organisations involved in strategy implementation well clarified and is there a common understanding of and agreement on how ‘successful implementation’ is to be defined and measured?  **A –** Does the strategy outline a comprehensive approach using multiple strategies at multiple levels targeting multiple population groups?  **D_2_ –** Are national PA guidelines widely disseminated and adapted according to different population groups? | **15.** Are the roles and responsibilities of organisations involved in strategy implementation well clarified and is there a common understanding of and agreement on how ‘successful implementation’ is to be defined and measured?  **16.** Does the strategy have a clear statement on the timeframe for policy implementation?  **17.** Does the strategy reference specific target groups?  **18.** Is the policy content predominantly ‘downstream’ or ‘upstream’?  **19.** Does the strategy outline a comprehensive approach using multiple strategies at multiple levels targeting multiple population groups? |
| **Effects:** Analysis of the economic, environmental, public health, social, and other potential impacts of policy. | Example questions (CAPPA):  -What kind of impact did the strategy have on walking levels?  -Were there any unintended consequences of the implementation of the strategy? | Proposed HARDWIRED questions:  **E** – Is there systematic surveillance of population levels of walking?  **I** – Is the evaluation conducted by an independent body which is not connected to the government or ‘policy owners’? | **20.** Is the evaluation conducted by an independent body which is not connected to the government or ‘policy owners’?  **21.** Is there systematic surveillance of population levels of walking?  **22.** What kind of impact did the strategy have on walking levels?  **23.** Were there any unintended consequences of the implementation of the strategy? |
