# Supplementary material for: A critical analysis of walking policy in Ireland and its contribution to both national and international development goals
Source: Front Sports Act Living. 2023 Mar 1;5:1125636. doi: 10.3389/fspor.2023.1125636 (PMC10014795; doi:10.3389/fspor.2023.1125636)
Supplement: Supplementary file 2 [file Table2.docx]

Supplementary file 2: Local level walking policies content analysis results

| **Scope of policy analysis section** | **Content analysis grid criteria** | **Cork: Cork City Walking Strategy 2013-2018** | **Carlow:** [**County Carlow’s Outdoor Recreation Strategy 2020-2023**](https://www.carlow.ie/wp-content/documents/uploads/Sli%202020%20-%20County%20Carlow%20Outdoor%20Recreation%20Strategy.pdf) | **Donegal:** [**North West Greenway Plan 2015**](https://www.derrystrabane.com/getmedia/56932767-0160-401e-a53a-84963957f617/North-West-Greenway-Plan_draft_5_15-10-15-reduced-size.pdf) | **Dublin:** [**The Heart of Dublin – City Centre Public Realm Master Plan 2016**](https://www.dublincity.ie/sites/default/files/2020-08/public-realm-masterplan.pdf) | **Longford:** [**County Longford Tourism Statement of Strategy and Work Programme 2017-2022**](https://www.longfordcoco.ie/services/community-development/documents/tourism/longford-tourism-strategy.pdf) | **Monaghan:** [**County Walking & Cycling Strategy 2021-2026**](https://monaghan.ie/wp-content/uploads/2021/10/Monaghan-Walking-Cycling-Strategy-adopted-by-Council-Oct-2021.pdf) | **Wexford:** [**County Wexford Tourism Strategy 2019-2023**](https://www.wexfordcoco.ie/sites/default/files/content/Business/Tourism%20Strategy%202019%20to%202023.pdf) | **Wicklow:** [**County Wicklow Recreation Strategy 2020-2025**](https://www.wicklow.ie/Portals/0/Documents/Community/Wiclow%20Outdoor%20Recreation%20Strategy/Co%20Wicklow%20Outdoor%20Recreation%20Strategy.pdf) |
| --- | --- | --- | --- | --- | --- | --- | --- | --- | --- |
| **Availability** | 1. Is there a national walking specific strategy for Ireland/[name of county]? | Yes. | No. | No. | No. | No. | No. | No. | No. |
| **Context** | **2.** What was the key stimulus for policy action? | County wide decline in the prevalence of walking for transport and increase in the use of private car for transport purposes. | Recognition of contribution of outdoor recreation to local economy. | Increasing demand for Greenway development in the North West Cross Border region. | Updating previous version of Public Realm Strategy for Dublin 2012. | To grow tourism to the county (p2). | <10% of people in Monaghan commute by walking or cycling. To increase walking for recreation and for tourism. | Economic benefits of tourism to local economy. | Update new strategy. Economic benefits of tourism to local economy. |
|  | **3.** Were local level strategies developed according to the separation of powers doctrine? | N/A | N/A | N/A | N/A | N/A | N/A | N/A | N/A |
|  | **4.** What budget was allocated for the implementation of the policy? | Not specified | Not specified. | Not specified. However, a list of funding sources from NI, ROI, cross-border, and EU sources are listed in section 6.1 (p74). | Not specified. | Not specified. | Not specified. Active Travel Unit mentioned. | Not specified. Page 104 suggested list of funding sources to support implementation. | Output 3.1 (p44) €20m external funding over the lifetime of the plan |
|  | **5.** Does the policy have a clear statement which is also embedded in other policy agendas? | CCWS linked with the following policies:  -Smarter Travel -DMURS -Cork City and County Development Plans -Unspecified regional policies -Unspecified national policies | Yes. Noted that this strategy is ‘framed’ by 11 local and national policies from multiple sectors including sport, health, tourism and commerce. | Yes. Page 32-33, provides objectives, vision, and  EU, NI and ROI policy contexts. | Clear statement of vision. No reference to connection to other policies. | Yes. Page 7 lists the broader policy context in which it sits. All tourism and regional development policies mentioned. | Yes. Section 2 (page8) contains a policy review of related national, regional, and local policies. | Yes. Section 2 (page 13) context at national and local level. | Clear statement of vision. Page 14 contextual analysis references policies from multiple sectors. |
| **Processes** | **6.** What process did the strategy have to go through to be implemented? | Stakeholder consultations were conducted with stakeholders from local government, health, the Garda Síochána, transport, academia, tourism, sport, roads, planning, environmental advocacy. Number of consultation unknown. | Public and individual meetings, online questionnaires and consumer surveys with individuals, clubs, organisation representatives. | Not specified. Sustrans lead the preparation of the document. | Survey, case studies, consultation. | Not specified. | Online submissions (presumably due to COVID). Page 32 mentions ‘consultation’ which involes online submissions. Appendix III (p59) contains list of submissions. | Interviews with 17 tourism and outdoor recreation stakeholders. 2 workshops and 1 world café with traders, industry partners and community members. | Consultation with n=700 stakeholders and community members through individual meetings, group meetings, public forums and online quesitonnaires. |
|  | **7.** Was a stakeholder analysis and needs assessment conducted to ensure widespread representation from interdisciplinary stakeholders at the early stages of strategy development? | Not stated. | Not stated. | Not stated. | Not stated. | SWOT Analysis conducted to identify a programme of work. | SWOT Analysis conducted. | Section 3 (p31) situational analysis and SWOT analysis conducted. | Not stated. |
|  | **8.** What mechanisms are in place to support the dissemination of the strategy? | Page 49 action a (ii) Establishing a communications strategy for the project. Presence unknown. | Action included on p34 outlining the development and publication of a communications strategy. | No. | Not specified. | Page 25 action e6.4.4. to develop a communications strategy to support the implementation of the plan | Not specified. | Page 102 – develop a communication plan. | Objecitve 2.7 – Review of Wicklow Outdoors online platform and plan; updated online strategy to profile outdoor recreation; a visual, data and marketing materials repository. |
| **Actors** | **9.** Does the strategy engage with grassroots practitioners, as well as policymakers, and define the organisational links between them? | Yes. No clarified definition of organisational links between stakeholders. | Yes. No clarified definition of organisational links between stakeholders. | Yes. No clarified definition of organisational links between stakeholders. | Yes. No clarified definition of organisational links between stakeholders. | Yes. No clarified definition of organisational links between stakeholders. | Yes. No clarified definition of organisational links between stakeholders. | Yes. No clarified definition of organisational links between stakeholders. | Yes. No clarified definition of organisational links between stakeholders. |
|  | **10.** What were the power relations between the actors involved in the development process? | Not specified. | Not specified. | Not specified. | Not specified. | Not specified. | Not specified. | Not specified. | Not specified. |
|  | **11.** Were actions within the strategy progressed through intersectoral partnerships? | Not specified. | Actions were assigned to multisectoral organisational teams. Progress unknown. Lack of representation from transport organisations. | Actions were assigned to multisectoral organisational teams. Progress unknown. | Not specified. | Actions were assigned to multisectoral organisational teams. Progress unknown. | Actions were assigned to multisectoral organisational teams. Progress unknown. | Actions were assigned to multisectoral organisational teams. Progress unknown. | Actions were assigned to multisectoral organisational teams. Progress unknown. |
| **Political will** | **12.** Did any political actor in power publicly express support to the development of the strategy? | Not specified. | Not specified. | Not specified. | Not specified. | Not specified. Local councillors provided foreword. | Not specified. | Not specified. | Not specified. |
|  | **13.** Is there a stable base of political and stakeholder support as well as sustained investment over the long term? | Not specified. | Not specified. | Not specified. | Not specified. | Not specified. | Not specified. | Not specified. | Not specified. |
|  | **14.** Does the government hold regular discussions with the aim to support the implementation of the strategy? | Not specified. | Not specified. | Not specified. | Not specified. | Not specified. | Not specified. | Not specified. | Not specified. |
| **Content** | **15.** Are the roles and responsibilities of organisations involved in strategy implementation well clarified and is there a common understanding of and agreement on how ‘successful implementation’ is to be defined and measured? | No. | Yes. Lead organisations and partners specified for each action and Key Performance Indicator outlined for each action. | Yes. Lead organisations and partners specified for each action, key Performance Indicator and estimated costs outlined for each action. | No. | No. Page 26 mentions a list of organisations. | Yes. Action plan (beginning p38) outlines responsibilities of each organisation to each action. Success and timelines also defined. | Yes (page 96). Organisations specified for each action, key Performance Indicator outlined for each action. | Yes(from p26). Organisations specified as lead or partners, actions and outputs defined. |
|  | **16.** Does the strategy have a clear statement on the timeframe for policy implementation? | Yes (2013-2018) | Yes (2020-2023) | No. | Yes (2016-2022) | Yes (2017-2022). | Yes (2021-2026) | Yes (2019-2023) | Yes (2020-2025) |
|  | **17.** Does the strategy reference specific target groups? | Yes. | Yes. | No. | Yes. | No. | Yes. | Yes. | Yes. |
|  | **18.** Is the policy content predominantly ‘downstream’ or ‘upstream’? | Unknown. | Combination. | Upstream. | Upstream. | Combination. | Downstream. | Combination. | Combination. |
|  | **19.** Does the strategy outline a comprehensive approach using multiple strategies at multiple levels targeting multiple population groups? | No. Majority of actions are within planning/urban design/transport sectors. | Yes. Lack of representation from transport organisations. | No. This strategy is focused on infrastructure development. | No. All actions within urban design/transport sector. | Unclear. | Yes. Lack of representation from academia and education. | Unclear. | Yes. |
| **Effects** | **20.** Is the evaluation conducted by an independent body which is not connected to the government or ‘policy owners’? | Not specified. | P33 Strategic Area ‘Research and Insights’ – lead partner IT Carlow (now SETU). | Not specified. | Not specified. | No. Monitoring of implementation of the strategy is the responsibility of lead organisations named on each action. | No. Page 51 Section Evaluation – note that ‘we’ (i.e. Monaghan County Council) will conduct evaluation. | Not specified. | Not specified. |
|  | **21.** Is there systematic surveillance of population levels of walking? | Not specified. | Not specified. | Not specified. | Not specified. | Not specified. | Not specified. | Not specified. | Not specified. |
|  | **22.** What kind of impact did the strategy have on walking levels? | Not specified. | Not specified. | Not specified. | Not specified. | Not specified. | Not specified. | Not specified. | Not specified. |
|  | **23.** Were there any unintended consequences of the implementation of the strategy? | Not specified. | Not specified. | Not specified. | Not specified. | Not specified. | Not specified. | Not specified. | Not specified. |
