# Supplementary material for: A critical analysis of walking policy in Ireland and its contribution to both national and international development goals
Source: Front Sports Act Living. 2023 Mar 1;5:1125636. doi: 10.3389/fspor.2023.1125636 (PMC10014795; doi:10.3389/fspor.2023.1125636)
Supplement: Supplementary file 3 [file Table3.docx]

Supplementary File 3: National Strategic Objectives and Get Ireland Walking Strategy and Action Plan 2017-2020: Conceptual linkage outcomes

| **National Strategic Objectives (NSO)** | **NSO Target Statement** | **Relevance to walking** | **Related GIW SAP 17-20 Actions** |
| --- | --- | --- | --- |
| **NSO 1: Compact Growth** | - 1. - Enable urban infill development that would not otherwise occur | Partially relevant | 4.2; 4.4 |
|  | 1.2 - Improve ‘liveability’ and quality of life, enabling greater densities of development to be achieved | Highly relevant | 4.1  4.6 |
|  | 1.3 - Encourage economic development and job creation, by creating conditions to attract internationally mobile investment and opportunities for indigenous enterprise growth | Partially relevant | 7.1 |
|  | 1.4 - Building on existing assets and capacity to create critical mass and scale for regional growth | Partially relevant | 4.1-4.6; 1.1-1.4 |
|  | 1.5 - Improve accessibility to and between centres of mass and scale and better integration with their surrounding areas | Highly relevant | 4.1-4.6 |
|  | 1.6 -Ensure transition to more sustainable modes of travel (walking, cycling, public transport) and energy consumption (efficiency, renewables) within an urban context | Highly relevant | 4.1-4.6 |
|  | 1.7 - Encourage labour mobility to support employment-led growth, including affordable housing, education/skills development and improved community and family services including childcare | Partially relevant | 4.4 |
|  | 1.8 -Enhance the attractiveness, viability and vibrancy of smaller towns and villages and rural areas as a means of achieving more sustainable patterns and forms of development | Highly relevant | 4.4 |
|  | 1.9 - Ensure transition to more sustainable modes of travel (walking, cycling, public transport) and energy consumption (efficiency, renewables) within smaller towns and villages and rural areas | Highly relevant | 5.8; 5.7 |
|  | 1.12 - Cross-boundary collaboration at county and regional level to achieve more sustainable outcomes for rural communities, e.g. applicable to shared settlements, landscapes and amenities as well as lands in state ownership | Partially relevant | 4.5; 4.4; 7.1 |
| **NSO 2: Enhanced Regional Accessibility** | 2.3 - Enabling more effective traffic management within and around cities and re-allocation of inner city road-space in favour of bus-based public transport services and walking/cycling facilities | Highly relevant | 4.1 |
|  | 2.8 - To strengthen public transport connectivity between cities and large growth towns in Ireland and Northern Ireland with improved services and reliable journey times | Partially relevant | 4.1 |
| **NSO 3: Strengthened Rural Economies and Communities** | 3.1 - Implementation of the actions outlined in the Action Plan for Rural Development | Partially relevant | 7.1 |
|  | 3.3 - Implementation of a targeted Rural Regeneration and Development Fund to enable opportunities to secure the rejuvenation and re-purposing of rural towns and villages weakened by the structural changes in rural economies and settlement patterns | Partially relevant | 7.1 |
|  | 3.4 - Provide a quality nationwide community based public transport system in rural Ireland which responds to local needs under the Rural Transport Network and similar initiatives | Partially relevant | 4.4 |
|  | 3.5 – Invest in maintaining regional and local roads and strategic road improvement projects in rural areas to ensure access to critical services such as education, healthcare and employment | Partially relevant | 4.4 |
|  | 3.6 - Invest in greenways, blueways and peatways as part of a nationally coordinated strategy | Highly relevant | 7.1 |
| **NSO 4: Sustainable mobility** | 4.1 - Expand attractive public transport alternatives to car transport to reduce congestion and emissions and enable the transport sector to cater for the demands associated with longer-term population and employment growth in a sustainable manner through the following measures | Highly relevant | 4.4; 7.1; 5.7; 5.8 |
|  | 4.2 - Deliver the key public transport objectives of the Transport Strategy for the Greater Dublin Area 2016-2035 by investing in projects such as New Metro Link, DART Expansion Programme, BusConnects in Dublin and key bus-based projects in the other cities and towns | Partially relevant | 4.1 |
|  | 4.3 - Provide public transport infrastructure and services to meet the needs of smaller towns, villages and rural areas | Partially relevant | 4.1 |
|  | 4.4 - Develop a comprehensive network of safe cycling routes in metropolitan areas to address travel needs and to provide similar facilities in towns and villages where appropriate | Partially relevant | 4.1 |
| **NSO 7: Enhanced Amenities and Heritage** | 7.1 - Implementation of planning and transport strategies for the five cities and other urban areas will be progressed with a major focus on improving walking and cycling routes, including continuous greenway networks and targeted measures to enhance permeability and connectivity | Highly relevant | 4.2; 7.1 |
|  | 7.2 - The Rural and Urban Regeneration and Development Funds will support transformational public realm initiatives to give city and town centre areas back to citizens, encouraging greater city and town centre living, enhanced recreational spaces and attractiveness from a cultural, tourism and promotional perspective | Highly relevant | 4.2; 7.1 |
|  | 7.3 - We will conserve, manage and present our heritage for its intrinsic value and as a support to economic renewal and sustainable employment | Partially relevant | 4.5 |
|  | 7.4 - Open up our heritage estates to public access, where possible | Highly relevant | 4.5; 4.3 |
|  | 7.5 - Invest in and enable access to recreational facilities, including trails networks, designed and delivered with a strong emphasis on conservation, allowing the protection and preservation of our most fragile environments and providing a wellbeing benefit for all | Highly relevant | 4.3; 4.5 |
| **NSO 10: Access to Quality Childcare, Education and Health Services** | 10.1 - Provide additional investment in the schools sector to keep pace with demographic demand and to manage increasing building and site costs so that new and refurbished schools on well-located sites within or close to existing built-up areas, can meet demographic growth and the diverse needs of local population | Highly relevant | 2.3; 2.2; |
|  | 10.2 - Expand and consolidate third-level facilities at locations where this will further strengthen the capacity of those institutions to deliver the talent necessary to drive economic and social development in the regions. The consolidation of the DIT campus at Grange Gorman is a critical flagship infrastructural project for the higher education sector | Partially relevant | 5.8; 6.3 |
