# Supplementary material for: A critical analysis of walking policy in Ireland and its contribution to both national and international development goals
Source: Front Sports Act Living. 2023 Mar 1;5:1125636. doi: 10.3389/fspor.2023.1125636 (PMC10014795; doi:10.3389/fspor.2023.1125636)
Supplement: Supplementary file 4 [file Table4.docx]

Supplementary File 4: Sustainable Development Goals and Get Ireland Walking Strategy and Action Plan 2017-2020: Conceptual linkage outcomes

| **Sustainable Development Goals** | **Sustainable Development Goal Targets** | **Related Get Ireland Walking Strategy and Action Plan 2017-2020** |
| --- | --- | --- |
| SDG 3 (Good Health and Wellbeing) | 3.4 By 2030, reduce by one third premature mortality from non-communicable diseases through prevention and treatment and promote mental health and well-being | 3.3; 5.1–5.4 |
| SDG 3 (Good Health and Wellbeing) | 3.5 Strengthen the prevention and treatment of substance abuse, including narcotic drug abuse and harmful use of alcohol | - |
| SDG 3 (Good Health and Wellbeing) | 3.6 By 2020, halve the number of global deaths and injuries from road traffic accidents | 4.2; 4.3; 4.4 |
| SDG 3 (Good Health and Wellbeing) | 3.9 By 2030, substantially reduce the number of deaths and illnesses from hazardous chemicals and air, water and soil pollution and contamination | 4.2; 4.4 |
| SDG 4 (Quality Education) | 4.7 By 2030, ensure that all learners acquire the knowledge and skills needed to promote sustainable development, including, among others, through education for sustainable development and sustainable lifestyles, human rights, gender equality, promotion of a culture of peace and non- violence, global citizenship and appreciation of cultural diversity and of culture’s contribution to sustainable development | 1.5; 1.6; 2.2 |
| SDG 8 (Decent Work and Economic Growth) | 8.1 Sustain per capita economic growth in accordance with national circumstances and, in particular, at least 7 per cent gross domestic product growth per annum in the least developed countries | 7.1 |
| SDG 8 (Decent Work and Economic Growth) | 8.9 By 2030, devise and implement policies to promote sustainable tourism that creates jobs and promotes local culture and products | 7.1 |
| SDG 11 (Sustainable Cities and Communities) | 11.a Support positive economic, social and environmental links between urban, peri-urban and rural areas by strengthening national and regional development planning | 4.2; 4.4 |
| SDG 11 (Sustainable Cities and Communities) | 11.2 By 2030, provide access to safe, affordable, accessible and sustainable transport systems for all, improving road safety, notably by expanding public transport, with special attention to the needs of those in vulnerable situations, women, children, persons with disabilities and older persons | 4.2; 4.4 |
| SDG 11 (Sustainable Cities and Communities) | 11.3 By 2030, enhance inclusive and sustainable urbanisation and capacity for participatory, integrated and sustainable human settlement planning and management in all countries" | 4.4 |
| SDG 11 (Sustainable Cities and Communities) | 11.6 By 2030, reduce the adverse per capita environmental impact of cities, including by paying special attention to air quality and municipal and other waste management | 4.2; 4.4 |
| SDG 11 (Sustainable Cities and Communities) | 11.7 By 2030, provide universal access to safe, inclusive and accessible, green and public spaces, in particular for women and children, older persons and persons with disabilities" | 4.2; 4.3; 4.4; 5.2 |
| SDG 12 (Responsible Consumption and Production) | 12.2 By 2030, achieve the sustainable management and efficient use of natural resources | - |
| SDG 12 (Responsible Consumption and Production) | 12.8 By 2030, ensure that people everywhere have the relevant information and awareness for sustainable development and lifestyles in harmony with nature | 1.1–1.4; 3.1–3.5; 4.1; 4.5 |
| SDG 13 (Climate Action) | 13.2 Integrate climate change measures into national policies, strategies and planning | - |
| SDG 16 (Peace, Justice and Strong Institutions) | 16.6 Develop effective, accountable and transparent institutions at all levels | 7.3 |
| SDG 16 (Peace, Justice and Strong Institutions) | 16.7 Ensure responsive, inclusive, participatory and representative decision- making at all levels | 7.1; 7.3 |
| SDG 17 (Partnerships for the Goals) | 17.16 Enhance the Global Partnership for Sustainable Development, complemented by multi-stakeholder partnerships that mobilise and share knowledge, expertise, technology and financial resources, to support the achievement of the SDGs in all countries, in particular developing countries | 7.1 |
| SDG 17 (Partnerships for the Goals) | 17.17 Encourage and promote effective public, public– private and civil society partnerships, building on the experience and resourcing strategies of partnerships" | 7.1; 7.3 |
